# Supplementary material for: Peptidoglycan DD-peptidases have distinct activities that impact fitness of Acinetobacter baumannii
Source: mBio. 2026 Mar 23;17(4):e00072-26. doi: 10.1128/mbio.00072-26 (PMC13059705; doi:10.1128/mbio.00072-26)
Supplement: Supplemental material — Figures S1-S7; Tables S1-S4. [file mbio.00072-26-s0001.docx]

**Supplemental data for ‘Peptidoglycan DD-peptidases have distinct activities that impact fitness of *Acinetobacter baumannii’***

Arshya Tehrani, Abhisha Khadka, Berenice Furlan, Michael Whalen, Jacob Biboy, Orietta Massidda, Waldemar Vollmer, Joseph M. Boll^#^

**Supplementary Figures**

**
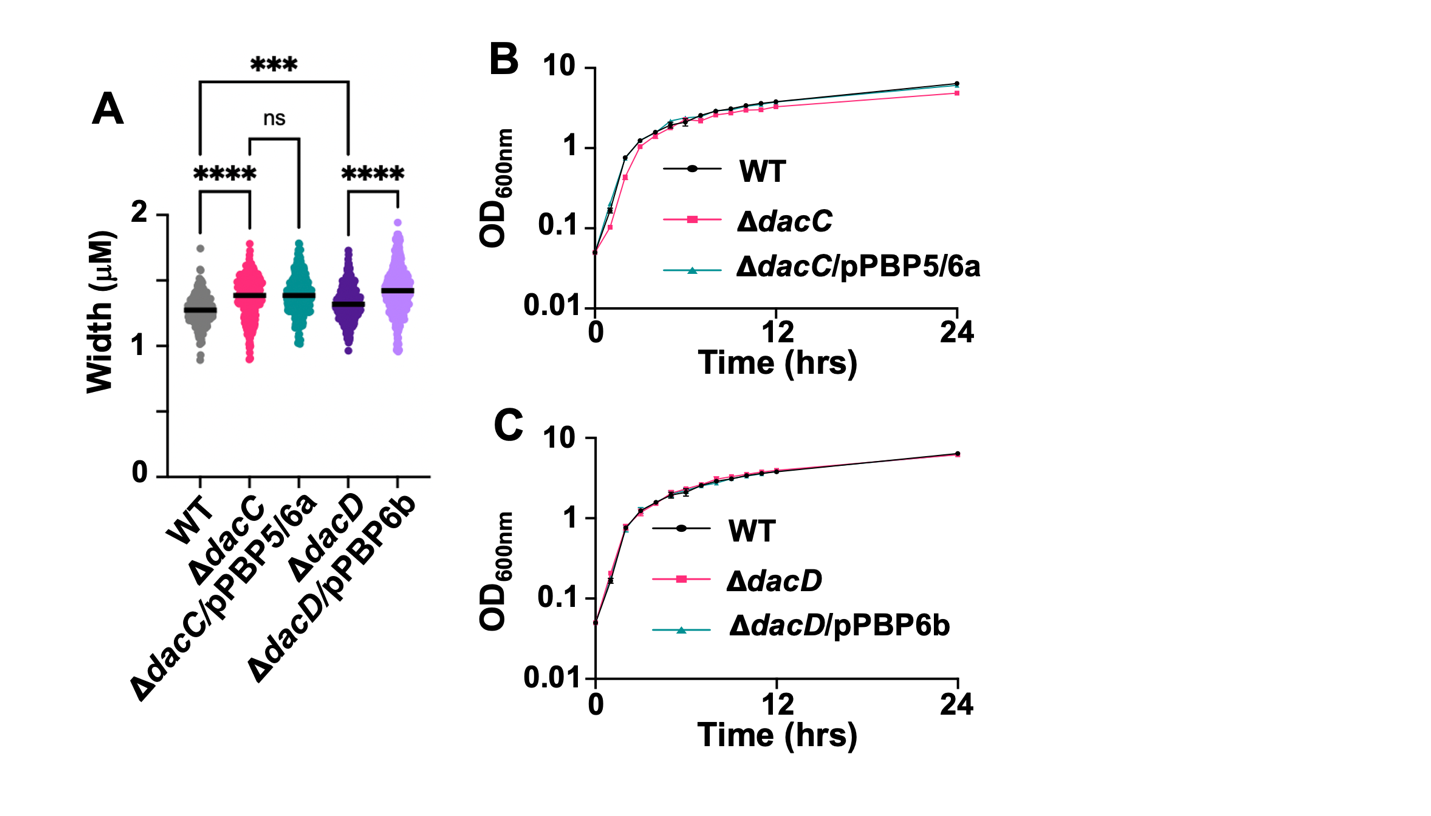
**

**Figure S1: Analysis of Δ*dacC* and Δ*dacD* mutations in *A. baumannii* strain ATCC 17978. (A)** Quantifications of cell width in wild-type (WT), ∆*dacC*, ∆*dacC*/pPBP5/6a, ∆*dacD*, and ∆*dacD*/pPBP6b (*n* >300), measured using ImageJ with the MicrobeJ plugin. Each dot represents a single cell. Error bars indicate standard deviation. Statistical significance was determined using one-way ANOVA (^***^ *P* < 0.001, ^****^ *P* < 0.0001, ns = not significant). **(B)** Growth curves of the Δ*dacC* mutant. **(C)** Growth curves of the Δ*dacD* mutant.


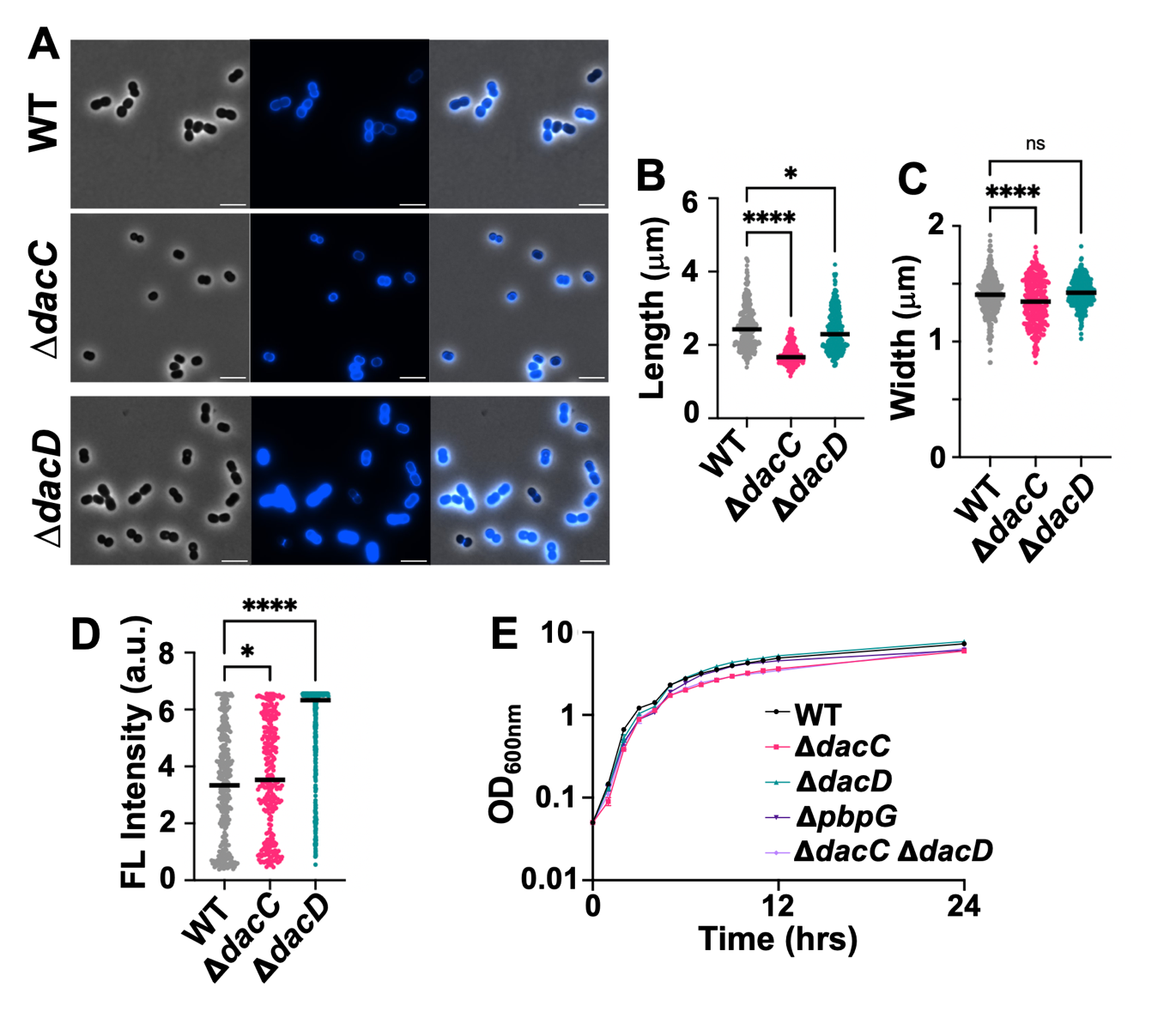


**Figure S2 : Analysis of *A. baumannii* strain AB5075 mutants. (A)** Phase-contrast (left) fluorescence (middle) and merged (right) images of wild-type (WT), ∆*dacC*, ∆*dacD* cells. Scale bar: 5 μm. (**B)** Quantification of cell length (pole to pole) for each strain (*n* >300) was measured using ImageJ with the MicrobeJ plugin. Each dot represents a single cell. Error bars indicate standard deviation. Statistical significance was determined using one-way ANOVA (^*^ *P* < 0.05, ^****^ *P* < 0.0001, ns = not significant). **(C)** Quantification of cell width. **(D)** Quantification of fluorescence intensity. **(E)** Growth curves of Δ*dacC,* Δ*dacD,* and Δ*pbpG* mutants. Each experiment was independently replicated three times; one representative data set is shown.


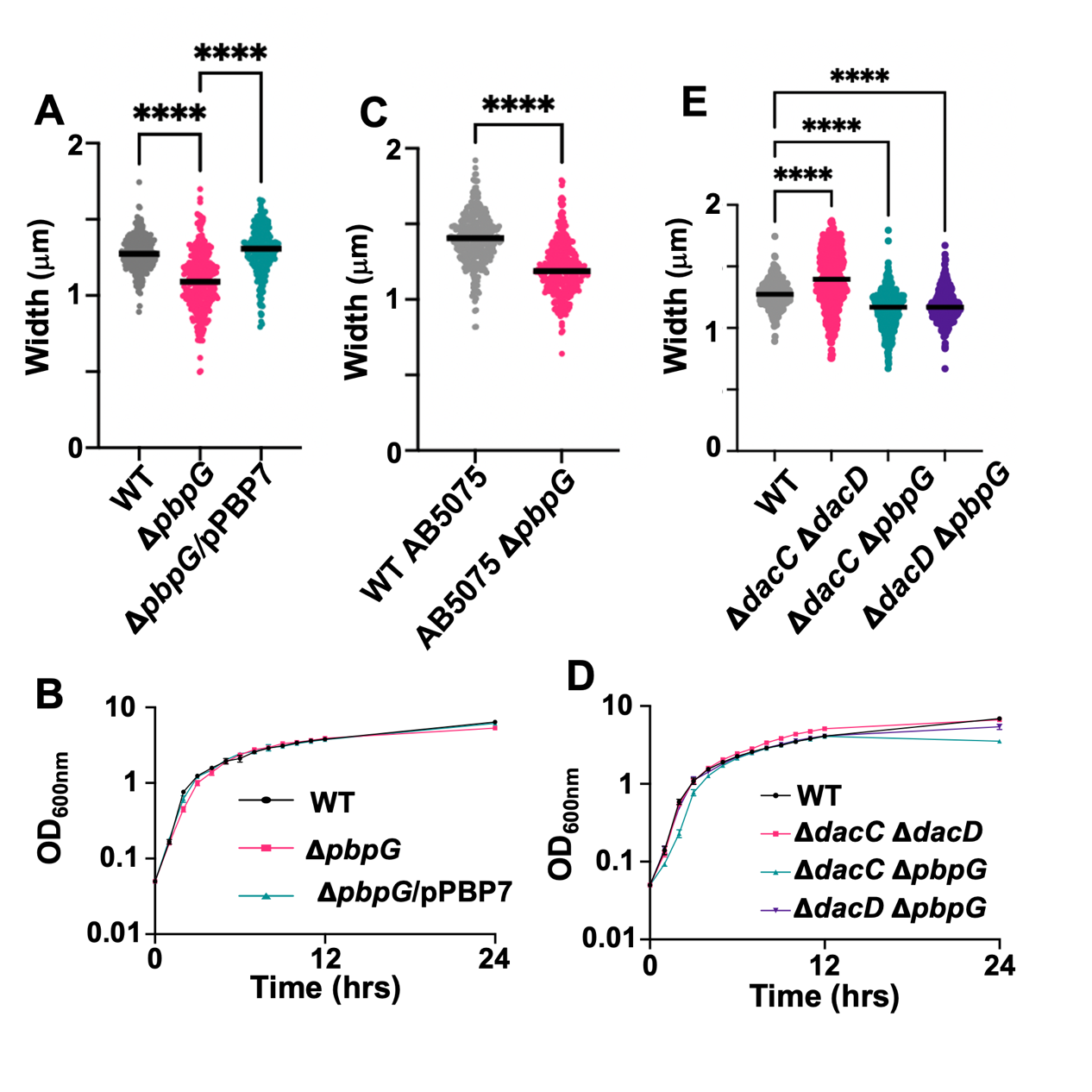


**Figure S3: Cell morphology measurement and growth curves of Δ*pbpG* mutants and double mutants. (A)** Quantifications of cell width in *A. baumannii* strain ATCC 17978 Δ*pbpG* mutants (*n* >300) , measured using ImageJ with the MicrobeJ plugin. Each dot represents a single cell. Error bars represent standard deviation. Statistical significance was determined using one-way ANOVA (^****^ *P* < 0.0001). **(B)** Growth curves of Δ*pbpG* mutants in strain ATCC 17978. **(C)** Same as described in (A), but for Δ*pbpG* mutants in *A. baumannii* strain AB5075. **(D)** Growth curves of double mutants in strain *A. baumannii* ATCC 17978. **(E)** Same as described in (A), but for double mutants in *A. baumannii* strain ATCC 17978.


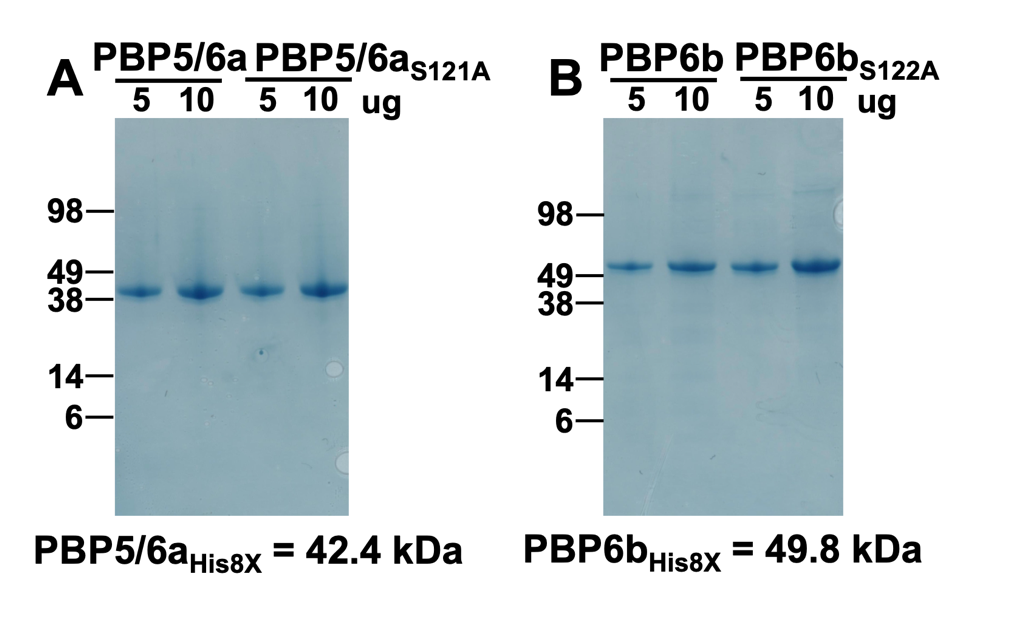


**Figure S4: Purification of PBP5/6a and PBP6b enzymes. (A)** Coomassie stained SDS-PAGE gel of PBP5/6a_His8X_ and the catalytically inactive mutant PBP5/6a_S121A His8X_. **(B)** Coomassie stained SDS-PAGE gel of PBP6b_His8X_ and the catalytically inactive mutant PBP6b_S122A His8X_.


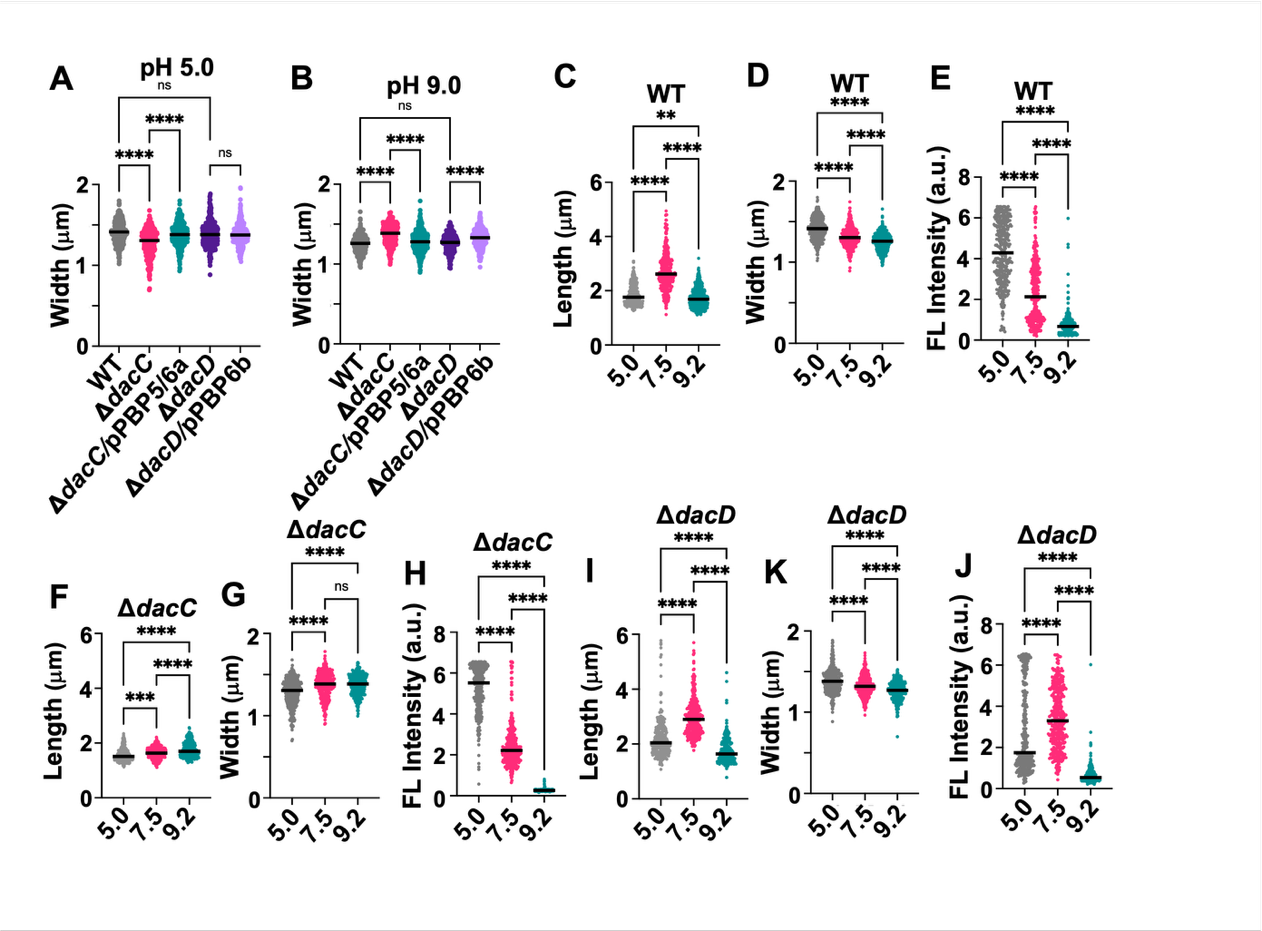


**Figure S5: Quantitative analysis of *A. baumannii* strain ATCC 17978 cell morphology and fluorescence under acidic and alkaline conditions. (A)** Quantifications of width in wild type (WT), ∆*dacC*, ∆*dacC*/pPBP5/6a, ∆*dacD*, and ∆*dacD*/pPBP6b strains grown at pH 5.0. **(B)** Same as (A), but for cells grown in pH 9.0. Quantifications (*n* >300) were performed using ImageJ with the MicrobeJ plugin. Each dot represents a single cell. Error bars represent standard deviation. Statistical significance was determined using one-way ANOVA (^**^ *P* <0.01, ^***^ *P* <0.001, ^****^ *P* <0.0001, ns = not significant). **(C)** Quantification of length, **(D)** width, and **(E)** fluorescence intensity in WT cells grown at pH 5.0, 7.5, and 9.0. **(F, G, H)** Same as described for (C-E), but for ∆*dacC* cells. **(I, J, K)** Same as described for (C-E), but for ∆*dacD* cells.


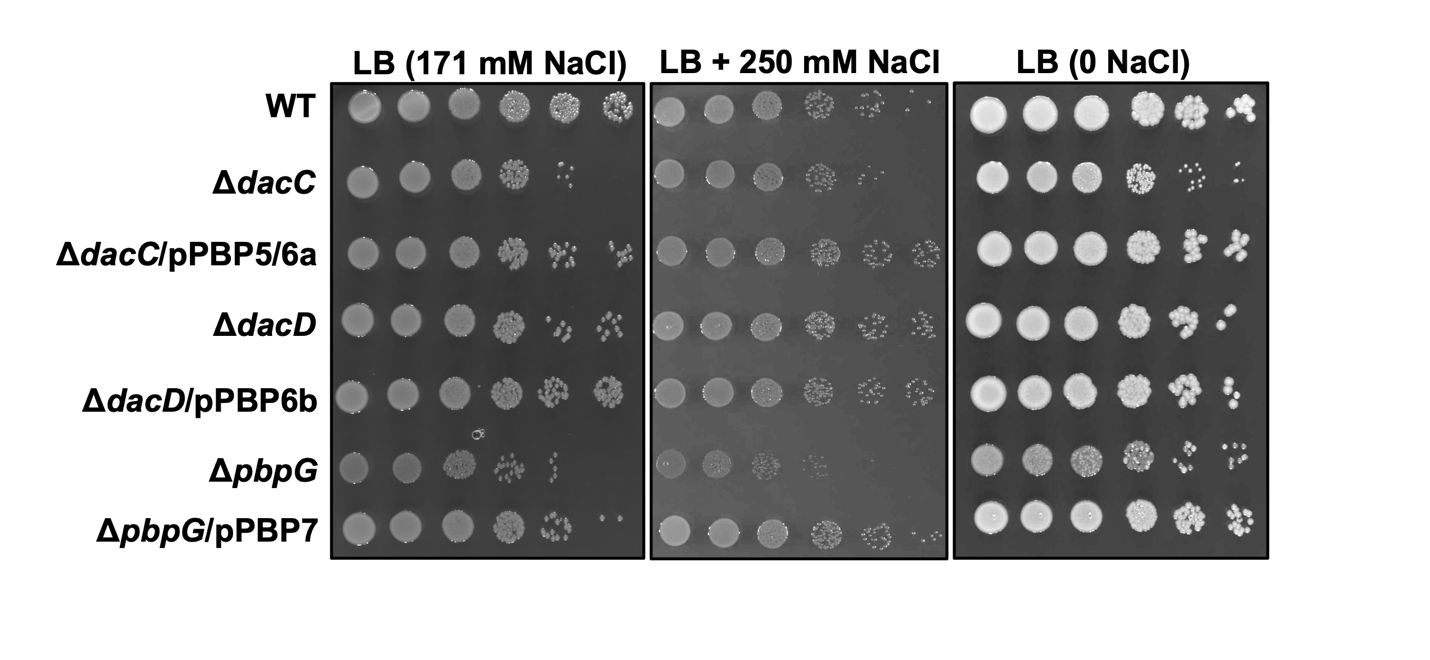
 **Figure S6: Colony growth of *A. baumannii* strain ATCC 17978 mutants under osmotic stress. (A)** Colony spot assays of wild-type (WT) and DD-CPase mutant strains. Cultures were serially diluted 10-fold starting at OD_600_ 0.05, plated on LB agar supplemented with the indicated NaCl concentrations to impose hypo- or hyperosmotic conditions.


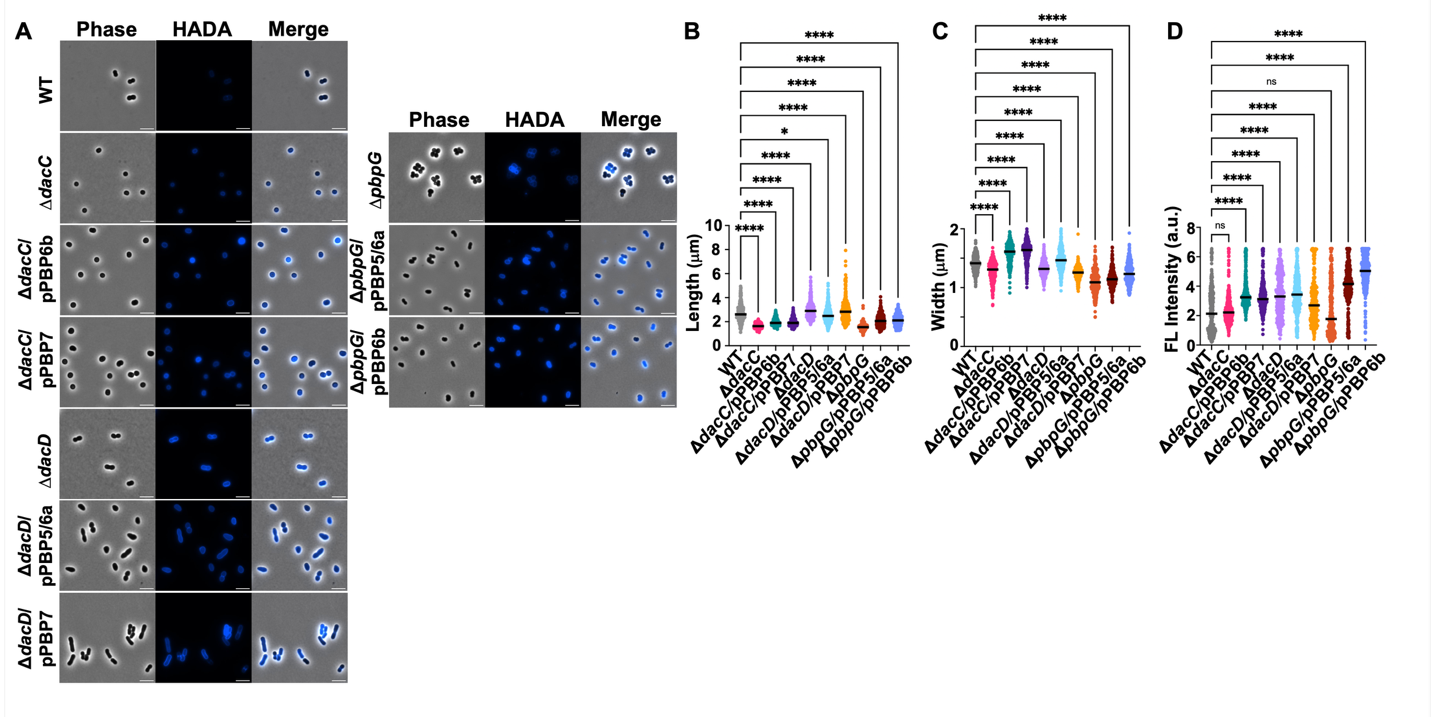


**Figure S7: DD-CPase mutants cannot be complemented by other DD-CPases. (A)** Representative phase-contrast (left), fluorescence (middle), and merged images of wild-type (WT), and DD-CPase mutants: ∆*dacC*, ∆*dacC*/pPBP6b, ∆*dacC*/pPBP7, ∆*dacD*, ∆*dacD*/pPBP5/6a, ∆*dacD*/pPBP7, ∆*pbpG,* ∆*pbpG*/pPBP5/6a, and ∆*pbpG*/pPBP6b. Scale bar: 5 μm. **(B)** Cell length measurements (pole to pole) for each strain (*n* > 300), quantified using ImageJ with the MicrobeJ plugin. Each dot represents a single cell; error bars indicate standard deviation. Statistical significance was determined using one-way ANOVA (*p* < 0.05, ns = not significant). **(C)** Quantifications of cell width. **(D)** Quantification of fluorescence intensity for the same cell populations. (a.u. - arbitrary units).

**Supplementary Tables**

**Table S1. Established DD-peptidases in *E. coli* *vs* homologs in *A. baumannii***

| ***E. coli*** | | | |  | ***A. baumannii*** | | | |
| --- | --- | --- | --- | --- | --- | --- | --- | --- |
| **Gene** | **Protein** | **Length (aa)** | **Function** |  | **Gene** | **Protein** | **Length (aa)** | **Predicted function** |
| *dacB* | PBP4 | 477 | DD-CPase; DD-EPase |  | n/d | n/d | n/d | - |
| *dacA* | PBP5 | 403 | DD-CPase |  | *dacC* | PBP5/6a | 382 | DD-CPase |
| *dacC* | PBP6a | 400 | DD-CPase |  |  |  |  |  |
| *dac*D | PBP6b | 388 | DD-CPase |  | *dacD* | PBP6b | 439 | DD-CPase; DD-EPase |
| *pbpG* | PBP7/8 | 479 | DD-EPase |  | *pbpG* | PBP7 | 348 | DD-CPase; DD-EPase |
| *ampC* | AmpC | 377 | -lactamase; DD-CPase |  | *bla_ADC-26_* | AmpC | 383 | -lactamase; DD-CPase? |
| *ampH* | AmpH/PBP4b | 385 | DD-CPase; DD-EPase |  | n/d | n/d | n/d | - |

DD-CPase: DD-carboxypeptidase

DD-EPase: DD-endopeptidase

n/d: no homolog detected

| **Table S2: Muropeptide composition of wild type (WT), Δ*dacC*, and Δ*dacD A. baumannii* strain ATCC 17978** | | | | | | | | | | | | | | |  |
| --- | --- | --- | --- | --- | --- | --- | --- | --- | --- | --- | --- | --- | --- | --- | --- |
| **Peak No.** | **Muropeptide** | **Relative % of each muropeptide^a^** | | | | | | | | | | | | |  |
|  |  | **WT Logarithmic** | **WT Stationary** | | **Δ*dacC* Logarithmic** | | | | **Δ*dacC* Stationary** | | **Δ*dacD* Logarithmic** | | **Δ*dacD* Stationary** | |  |
| **1** | **Tri** | 2.5 ± 0.0 | 2.9 ± 0.0 | | 3.3 ± 0.2 | | | | 4.0 ± 0.1 | | 2.6 ± 0.0 | | 2.9 ± 0.1 | |  |
| **2** | **Tri-D-Asn** | 0.7 ± 0.0 | 0.4 ± 0.0 | | 0.7 ± 0.0 | | | | 0.6 ± 0.0 | | 0.5 ± 0.0 | | 1.1 ± 2.2 | |  |
| **3** | **Tri-D-Lys** | 0.1 ± 0.0 | 0.7 ± 0.1 | | 0.5 ± 0.0 | | | | 1.2 ± 1.0 | | 0.7 ± 0.0 | | 1.5 ± 1.3 | |  |
| **4** | **TetraGly4** | 1.8 ± 0.0 | 1.5 ± 0.0 | | 2.6 ± 0.0 | | | | 1.3 ± 0.8 | | 1.6 ± 0.0 | | 1.9 ± 3.1 | |  |
| **5** | **Tetra-D-Lys** | 0.0 ± 0.0 | 0.2 ± 0.1 | | 0.0 ± 0.0 | | | | 0.0 ± 0.0 | | 0.4 ± 0.3 | | 0.0 ± 0.0 | |  |
| **6** | **Tetra** | 19.0 ± 0.4 | 20.7 ± 0.0 | | 16.9 ± 7.6 | | | | 19.1 ± 0.1 | | 16.0 ± 0.4 | | 17.6 ± 0.0 | |  |
| **7** | **Tetra-D-Arg** | 0.0 ± 0.0 | 0.3 ± 0.0 | | 0.0 ± 0.0 | | | | 0.4 ± 0.0 | | 0.5 ± 0.0 | | 1.5 ± 0.0 | |  |
| **7B** | **Penta** | 0.0 ± 0.0 | 0.0 ± 0.0 | | 0.5 ± 0.0 | | | | 0.7 ± 0.1 | | 0.1 ± 0.0 | | 0.0 ± 0.0 | |  |
| **8** | **TetraTriDapGly4** | 0.2 ± 0.0 | 0.4 ± 0.0 | | 0.5 ± 0.0 | | | | 0.3 ± 0.1 | | 0.1 ± 0.0 | | 0.1 ± 0.1 | |  |
| **9** | **TriTri(Dap)/TriTriDap-D-Lys** | 0.5 ± 0.0 | 0.9 ± 0.1 | | 0.6 ± 0.0 | | | | 1.3 ± 0.1 | | 0.5 ± 0.0 | | 0.8 ± 0.2 | |  |
| **10** | **TetraTri(Dap)** | 0.0 ± 0.0 | 0.2 ± 0.1 | | 0.3 ± 0.1 | | | | 0.4 ± 0.0 | | 0.2 ± 0.0 | | 0.8 ± 1.1 | |  |
| **11** | **TetraTri** | 3.7 ± 0.1 | 4.0 ± 0.2 | | 4.0 ± 0.1 | | | | 4.9 ± 0.6 | | 3.1 ± 0.0 | | 3.7 ± 0.7 | |  |
| **12** | **TetraTri-D-Lys** | 0.3 ± 0.1 | 1.9 ± 0.7 | | 1.5 ± 0.6 | | | | 2.5 ± 2.0 | | 0.9 ± 0.3 | | 1.9 ± 1.8 | |  |
| **13** | **TetraTri-D-Lys** | 0.9 ± 0.1 | 1.0 ± 0.6 | | 0.4 ± 0.2 | | | | 1.6 ± 0.2 | | 0.4 ± 0.0 | | 1.6 ± 0.4 | |  |
| **14** | **TetraTri-D-Arg** | 0.5 ± 0.0 | 1.0 ± 0.4 | | 1.4 ± 0.4 | | | | 1.1 ± 1.3 | | 2.1 ± 0.1 | | 3.4 ± 19.2 | |  |
| **15** | **TetraTetra** | 37.7 ± 1.4 | 31.5 ± 1.0 | | 32.5 ± 5.8 | | | | 25.8 ± 0.8 | | 34.7 ± 0.6 | | 25.4 ± 24.5 | |  |
| **15B** | **TetraPenta** | 0.4 ± 0.0 | 0.7 ± 0.1 | | 1.6 ± 0.0 | | | | 1.8 ± 0.0 | | 1.6 ± 0.4 | | 2.8 ± 0.0 | |  |
| **16** | **TetraTetraTri or TetraTetraTriDap** | 1.1 ± 0.0 | 1.7 ± 0.0 | | 1.0 ± 0.5 | | | | 2.0 ± 0.0 | | 1.1 ± 0.0 | | 1.4 ± 0.0 | |  |
| **17** | **TetraTetraTri or TetraTetraTriDap** | 0.2 ± 0.0 | 0.6 ± 0.1 | | 0.8 ± 0.2 | | | | 1.7 ± 1.0 | | 0.6 ± 0.1 | | 0.6 ± 0.1 | |  |
| **18** | **TriTriDap-D-Met** | 0.8 ± 0.0 | 1.1 ± 0.2 | | 1.0 ± 0.8 | | | | 0.7 ± 0.2 | | 0.3 ± 0.2 | | 1.1 ± 0.1 | |  |
| **19** | **TetraTetraTetra** | 18.5 ± 0.0 | 16.1 ± 0.1 | | 15.2 ± 7.2 | | | | 12.8 ± 0.0 | | 17.8 ± 0.0 | | 13.6 ± 0.1 | |  |
| **20** | **TetraTri-D-Met** | 0.4 ± 0.0 | 0.5 ± 0.0 | | 0.6 ± 0.0 | | | | 1.0 ± 0.1 | | 0.3 ± 0.0 | | 0.5 ± 0.1 | |  |
| **21** | **TetraTriAnh / TetraTetraTetraTri** | 4.8 ± 0.0 | 4.2 ± 0.0 | | 3.7 ± 1.8 | | | | 3.3 ± 0.0 | | 4.7 ± 0.0 | | 3.5 ± 0.1 | |  |
| **22** | **TetraTetraAnh I** | 1.7 ± 0.2 | 1.3 ± 0.1 | | 1.8 ± 0.8 | | | | 1.1 ± 0.2 | | 1.6 ± 0.3 | | 1.2 ± 0.2 | |  |
| **23** | **TetraTetraAnh II** | 0.7 ± 0.0 | 0.9 ± 0.0 | | 0.8 ± 0.0 | | | | 0.9 ± 0.0 | | 0.7 ± 0.0 | | 0.9 ± 0.1 | |  |
| **24** | **TetraTetraTetraAnh** | 2.0 ± 0.0 | 2.1 ± 0.0 | | 2.0 ± 0.0 | | | | 1.7 ± 0.1 | | 2.1 ± 0.0 | | 2.0 ± 0.0 | |  |
| **Sum of known peaks** | | 97.7 ± 1.1 | 96.0 ± 3.9 | | 93.2 ± 27.4 | | | | 91.5 ± 0.0 | | 94.7 ± 0.7 | | 90.9 ± 0.3 | |  |
|  |  |  |  | |  | | | |  | |  | |  | |  |
| **Monomers (Total)** | | 24.5 ± 0.7 | 27.7 ± 0.0 | | 26.2 ± 19.8 | | | | 29.6 ± 0.0 | | 23.5 ± 0.0 | | 28.9 ± 5.4 | |  |
| **Monomers with modification** | | 2.6 ± 0.0 | 3.2 ± 0.0 | | 4.0 ± 0.0 | | | | 3.7 ± 0.2 | | 3.8 ± 0.1 | | 6.4 ± 4.5 | |  |
| **Monomer tri** | | 2.5 ± 0.0 | 3.0 ± 0.0 | | 3.5 ± 0.5 | | | | 4.4 ± 0.1 | | 2.7 ± 0.1 | | 3.2 ± 0.1 | |  |
| **Monomer tri-D-Asn** | | 0.7 ± 0.0 | 0.4 ± 0.0 | | 0.8 ± 0.0 | | | | 0.6 ± 0.0 | | 0.5 ± 0.0 | | 1.1 ± 2.4 | |  |
| **Monomer tri-D-Lys** | | 0.1 ± 0.0 | 0.7 ± 0.1 | | 0.5 ± 0.0 | | | | 1.3 ± 1.3 | | 0.7 ± 0.0 | | 1.7 ± 1.6 | |  |
| **Monomer tetraGly4** | | 1.9 ± 0.0 | 1.5 ± 0.0 | | 2.7 ± 0.0 | | | | 1.4 ± 1.0 | | 1.7 ± 0.0 | | 2.1 ± 3.6 | |  |
| **Monomer tetra-D-Lys** | | 0.0 ± 0.0 | 0.3 ± 0.1 | | 0.0 ± 0.0 | | | | 0.0 ± 0.0 | | 0.4 ± 0.3 | | 0.0 ± 0.0 | |  |
| **Monomer tetra** | | 19.4 ± 0.7 | 21.5 ± 0.1 | | 18.2 ± 15.7 | | | | 20.9 ± 0.2 | | 16.9 ± 0.2 | | 19.3 ± 0.0 | |  |
| **Monomer tetra-D-Arg** | | 0.0 ± 0.0 | 0.3 ± 0.0 | | 0.0 ± 0.0 | | | | 0.4 ± 0.0 | | 0.6 ± 0.0 | | 1.6 ± 0.0 | |  |
| **Monomer penta** | | 0.0 ± 0.0 | 0.0 ± 0.0 | | 0.5 ± 0.0 | | | | 0.8 ± 0.1 | | 0.1 ± 0.0 | | 0.0 ± 0.0 | |  |
|  |  |  |  | |  | | | |  | |  | |  | |  |
| **Dimers (Total)** | | 53.2 ± 1.0 | 51.3 ± 0.2 | | 53.7 ± 6.1 | | | | 50.7 ± 0.6 | | 53.9 ± 0.0 | | 52.0 ± 5.4 | |  |
| **Dimers with modification** | | 3.4 ± 0.2 | 6.9 ± 0.0 | | 6.0 ± 3.4 | | | | 9.1 ± 0.2 | | 4.9 ± 0.1 | | 10.1 ± 8.4 | |  |
| **Dimer chain ends (anhydroMur*N*Ac)** | | 7.3 ± 0.5 | 6.5 ± 0.1 | | 6.6 ± 0.0 | | | | 5.7 ± 0.2 | | 7.4 ± 0.4 | | 6.1 ± 0.2 | |  |
|  |  |  |  | |  | | | |  | |  | |  | |  |
| **Trimers (Total)** | | 22.3 ± 0.0 | 21.2 ± 0.0 | | 20.3 ± 4.2 | | | | 19.9 ± 0.6 | | 22.8 ± 0.0 | | 19.2 ± 0.0 | |  |
| **Trimer chain ends (anhydroMur*N*Ac)** | | 2.1 ± 0.0 | 2.1 ± 0.0 | | 2.2 ± 0.0 | | | | 1.9 ± 0.1 | | 2.3 ± 0.0 | | 2.2 ± 0.0 | |  |
|  |  |  |  | |  | | | |  | |  | |  | |  |
| **Tripeptides (Total)** | | 10.0 ± 0.1 | 13.0 ± 0.3 | | 12.9 ± 1.8 | | | | 17.0 ± 4.5 | | 11.2 ± 0.1 | | 16.5 ± 3.4 | |  |
| **Tripeptides with modifications** | | 3.1 ± 0.1 | 5.6 ± 0.4 | | 5.1 ± 2.2 | | | | 7.5 ± 1.0 | | 4.1 ± 0.0 | | 8.8 ± 1.6 | |  |
| **Tetrapeptides (Total)** | | 89.5 ± 0.1 | 86.0 ± 0.7 | | 85.3 ± 2.4 | | | | 80.5 ± 3.9 | | 87.6 ± 0.0 | | 81.5 ± 2.4 | |  |
| **Tetrapeptides with modifications** | | 2.9 ± 0.1 | 4.5 ± 0.4 | | 4.9 ± 0.2 | | | | 5.3 ± 1.0 | | 4.7 ± 0.2 | | 7.7 ± 14.0 | |  |
| **Pentapeptides** | | 0.2 ± 0.0 | 0.3 ± 0.0 | | 1.3 ± 0.0 | | | | 1.7 ± 0.0 | | 1.0 ± 0.0 | | 1.5 ± 0.0 | |  |
|  |  |  |  | |  | | | |  | |  | |  | |  |
| **3-3 Crosslinks** | | 1.1 ± 0.0 | 2.1 ± 0.0 | | 1.8 ± 0.6 | | | | 2.8 ± 0.0 | | 1.2 ± 0.1 | | 2.2 ± 0.1 | |  |
| **Chain ends (anhydroMur*N*Ac)** | | 4.3 ± 0.1 | 4.0 ± 0.0 | | 4.0 ± 0.0 | | | | 3.5 ± 0.1 | | 4.4 ± 0.1 | | 3.8 ± 0.0 | |  |
|  |  |  |  | |  |  | | | |  | |  | |  | |
| **Degree of crosslinkage** | | 41.4 ± 0.2 | 39.7 ± 0.0 | | 40.3 ± 6.5 | | | | 38.6 ± 0.0 | | 42.1 ± 0.0 | | 38.8 ± 1.4 | |  |
| **% peptides in cross-links** | | 75.5 ± 0.7 | 72.4 ± 0.0 | | 73.9 ± 19.8 | | | | 70.5 ± 0.0 | | 76.6 ± 0.0 | | 71.2 ± 5.4 | |  |
| **^a^Values are mean ± variation of two biological repeats.** | | |  |  | | |  |  |  |  |  |  |  |  |  |

| **Table S3: Strains and plasmids used in this study**. | | |
| --- | --- | --- |
| **Strain/Plasmid** | **Description** | **Reference/Source** |
| **Strains** |  |  |
| *E. coli* C2987 | chemically competent wild type, K-12 | New England Biolabs |
| *E. coli* C2527 | chemically competent BL-21 | New England Biolabs |
| *A. baumannii* ATCC 17978 | wild type | ATCC (1) |
| *A. baumannii* 5075 | wild type | (2) |
| *A. baumannii* ATCC 17978 | Δ*dacC* | This Study |
| *A. baumannii* ATCC 17978 | Δ*dacC*/pPBP5/6a | This Study |
| *A. baumannii* ATCC 17978 | Δ*dacC*/pPBP6b | This Study |
| *A. baumannii* ATCC 17978 | Δ*dacC*/pPBP7 | This Study |
| *A. baumannii* ATCC 17978 | Δ*dacD* | This Study |
| *A. baumannii* ATCC 17978 | Δ*dacD*/pPBP6b | This Study |
| *A. baumannii* ATCC 17978 | Δ*dacD*/pPBP5/6a | This Study |
| *A. baumannii* ATCC 17978 | Δ*dacD*/pPBP7 | This Study |
| *A. baumannii* ATCC 17978 | Δ*pbpG* | (3) |
| *A. baumannii* ATCC 17978 | Δ*pbpG*/pPBP7 | (3) |
| *A. baumannii* ATCC 17978 | Δ*pbpG*/pPBP5/6a | This Study |
| *A. baumannii* ATCC 17978 | Δ*pbpG*/pPBP6b | This Study |
| *A. baumannii* ATCC 17978 | Δ*dacC* Δ*dacD* | This Study |
| *A. baumannii* ATCC 17978 | Δ*dacC* Δ*pbpG* | This Study |
| *A. baumannii* ATCC 17978 | Δ*dacD* Δ*pbpG* | This Study |
| *A. baumannii* 5075 | Δ*dacC* Δ*dacD* | This Study |
| *A. baumannii* 5075 | Δ*dacC* Δ*pbpG* | This Study |
| *A. baumannii* 5075 | Δ*dacD* Δ*pbpG* | This Study |
| **Plasmids** |  |  |
| pABBRKn | pABBR_MCS with the *Kan^R^* gene from pKD4 inserted into the PvuI site, Kn^R^ | (5) |
| pAT03 | pMMB67EH with FLP recombinase, Amp^R^ | (4) |
| pAT04 | pMMB67EH with REC_Ab_ system, Tet^R^ | (4) |
| pKD4 | Kan^R^ | (6) |
| pT7-7 | Amp^R^ | (7) |
| pT7-7Kn | pT7-7 with the *Kan^R^* gene from pKD4 inserted into the PvuI site, Kn^R^ | (3) |
| pPBP5/6a-His_8X_ | pT7-7 with *dacC* (A1S_2435) cloned into the NdeI and BamHI sites, Kn^R^ | This study |
| pPBP5/6a_S121A_-His_8X_ | pT7-7 with *dacC*_S121A_ cloned into the NdeI and BamHI sites, Kn^R^ | This study |
| pPBP7 | pMMB67EHKn with the *pbpG* (A1S_0237) gene and native promoter inserted into the XhoI and KpnI sites, Kn^R^ | (3) |
| pPBP6b-His_8X_ | pT7-7 with *dacD* (A1S_2479) cloned into the NdeI and BamHI sites, Kn^R^ | This study |
| pPBP6b_S122A_-His_8X_ | pT7-7 with *dacD* _S122A_ cloned into the NdeI and BamHI sites, Kn^R^ | This study |
| pPBP5/6a | (A1S_2435) gene and native promoter inserted into the XhoI and KpnI sites, Kn^R^ | This study |
| pPBP6b | (A1S_2479) gene and native promoter inserted into the XhoI and KpnI sites, Kn^R^ | This study |
|  |  |  |

| **Table S4: Oligonucleotides used in this study.** | |
| --- | --- |
| **Oligo Name** | **Sequence (5’ to 3’)** |
| **Deletion Primers** |  |
| 17978 *dacC* Kan-FRT 5’ | GTTAAAAACTCTCTAGTTACAATACTGTTTGAAAAAGCCGACTTCCCCCATAGAAGTCGGCTTTGCTTTATCCTAAAATGCTAGGCTCTTCTGCTTCATACAAGATATTGGAATTACCTAGAATGATATCCTCCTTAGTTCCTATTCCG |
| 17978 *dacC* Kan-FRT 3’ | AACTTTACCATCTAAACTTGCAACAAGCTTACCAACGACTTGACCTTTTTGAAGTGGTGCATTTAGGTTCGGTTGAACAACCAATTGAGTTTTAATGCCGTCCGCTTTGCCTTTAGGCATAGTTAAGCGATTGTGTAGGCTGGAGCTGCTTCG |
| 17978 *dacC*-kan verify 5’ | AGCACGTAATGATGCAAAAGCTG |
| 17978 *dacC*-kan verify 3’ | CTTGGATCAATTTGTGGGTGATGGTC |
| 17978 *dacD* Kan-FRT 5’ | TTTGTATCGACTTGCGTAGATGTATTTTTTGGCAACAATTCACACTAGCCTTCTACAAAAAAAAGGCATAGCATGCTGGGCATCTGTGTTCCGTATTTGCCAGTAAACGTCAAGGTCACCTAGTG |
| 17978 *dacD* Kan-FRT 3’ | TTTGCTTCTTCAATGTGTACATCATTTTCAATTTGAAGACTGCGAATGAGCTGGTTGTTTTGATAAATTGAAACTGTTGCCAAATTCATCGCTTTCATTAACGGTGCTGTTAACTTTTGCTCATT |
| 17978 *dacD*-kan verify 5’ | GCGAATTGTCACGTGAACAAGG |
| 17978 *dacD*-kan verify 3’ | GCGTGGCGAGTTCTAAACCAC |
| 17978 *pbpG*-Kan FRT 5’ | AAAGCTTTATACCTTATATCTCAAATGTAAGGCATAATGATAGTAAGCGCAAATGTGTGTCACCCTGAGTCGAGTATTGTGCCGTGAAAAATTCTAAAAAGTCTTTAATGCATGTGCTAAGCATGatatcctccttagttcctattccg |
| 17978 *pbpG* -Kan FRT 3’ | TATCTAATATGTAAAATCTGAGTTTTTATAAAAAGCGGCTGTTTAATACAGCCGTTTTTTTATGCTTTTTAAATGGCATAAAAAAACGCTTCTTAAAAGAAGCGTTTTTAAAAATAATTAAATTAagcgattgtgtaggctggagctgcttcg |
| 17978 *pbpG*-kan verify 5’ | GCTTGCAATGGAATGACAAAATTAGCAATC |
| 17978 *pbpG*-kan verify 3’ | GATACAGCAATTAAACAATGTGCTGATGCAG |
| **Complementation Primers** |  |
| pABBRKn-*dacC*-F XhoI | CGCCTCGAGatgcaaaagctgctggtaacgaac |
| pABBRKn-*dacC*-R KpnI | CGCGGTACCccacggacatttcatcaatgtaacagc |
| pABBRKn-*dacD*-F XhoI | CGCCTCGAGttcctgatttggattcgctaaaaagcg |
| pABBRKn-*dacD*-R KpnI | CGCGGTACCggctttaccatttttcccaaaagtttcacc |
| pABBRKn-*pbpG*-F XhoI | AAATTACTCGAGCTATTCTTCTATAGTGAGCGAATAGTTG |
| pABBRKn-*pbpG*-R KpnI | GTTGTCGGTACCTGCAACAATGGACCAAGTAAAAGATTCG |
| **Overexpression Primers** |  |
| pT7-7::*dacC* NdeI | CGCCATATGACTCGAAAAAGCGCTATTGCTGCACTCCTCCTCTTAC |
| pT7-7::*dacC* BamHI 8X-his | CGCGGATCCTTAATGGTGATGGTGATGGTGATGGTGGTTGCTGAAGAATTGTTTGATATGG |
| pT7-7::*dacD* NdeI | CGCCATATGAAATTCTTCCTATCTCTTTTTACGCTGTTTAGTATTTTCTGTACTACTCTTACC |
| pT7-7::*dacD* BamHI 8X-his | CGCGGATCCTTAATGGTGATGGTGATGGTGATGGTGGTGCGAATCTATAGGG |
| **Sequencing Primers** |  |
| pABBRKn confirm 1 | GGGCTGACCGCTTCCT |
| pABBRKn confirm 2 | CGCTAGCAGCACGCCATAG |

**References**

1. Baumann P, Doudoroff M, Stanier RY. 1968. A study of the *Moraxella* group. II. Oxidative-negative species (genus *Acinetobacter*). *J Bacteriol* 95:1520–1541.

2. Gallagher LA, Ramage E, Weiss EJ, Radey M, Hayden HS, Held KG, Huse HK, Zurawski DV, Brittnacher MJ, Manoil C. 2015. Resources for Genetic and Genomic Analysis of Emerging Pathogen *Acinetobacter baumannii. J Bacteriol* 197:2027–2035.

3. Islam N, Kazi MI, Kang KN, Biboy J, Gray J, Ahmed F, Schargel RD, Boutte CC, Dörr T, Vollmer W, Boll JM. 2022. Peptidoglycan Recycling Promotes Outer Membrane Integrity and Carbapenem Tolerance in *Acinetobacter baumannii. mBio* 13:e0100122.

4. Tucker AT, Nowicki EM, Boll JM, Knauf GA, Burdis NC, Trent MS, Davies BW. 2014. Defining gene-phenotype relationships in *Acinetobacter baumannii* through one-step chromosomal gene inactivation. *mBio* 5:e01313-01314.

5. Boll JM, Crofts AA, Peters K, Cattoir V, Vollmer W, Davies BW, Trent MS. 2016. A penicillin-binding protein inhibits selection of colistin-resistant, lipooligosaccharide-deficient *Acinetobacter baumannii. Proc Natl Acad Sci U S A* 113:E6228–E6237.

6. Datsenko KA, Wanner BL. 2000. One-step inactivation of chromosomal genes in *Escherichia coli* K-12 using PCR products. *Proc Natl Acad Sci U S A* 97:6640–6645.

7. Studier FW, Rosenberg AH, Dunn JJ, Dubendorff JW. 1990. Use of T7 RNA polymerase to direct expression of cloned genes. *Methods Enzymol* 185:60–89.
